# “Owl's eyes” sign in acute spinal cord infarction in newborn submitted to aortoplasty

**DOI:** 10.1055/s-0042-1758392

**Published:** 2022-12-28

**Authors:** Arthur Cesario de Holanda, Mayllin Freitas Nunes, Fabiola Lys de Medeiros, Eduardo Sousa de Melo

**Affiliations:** 1 Universidade Federal de Pernambuco, Hospital das Clínicas, Unidade de Neurologia e Neurocirurgia, Recife PE, Brazil.; 2 Universidade de Pernambuco, Hospital Universitário Oswaldo Cruz, Recife PE, Brazil.; 3 Universidade Federal de Pernambuco, Centro de Ciências Médicas, Recife PE, Brazil.

**Figure 1:**
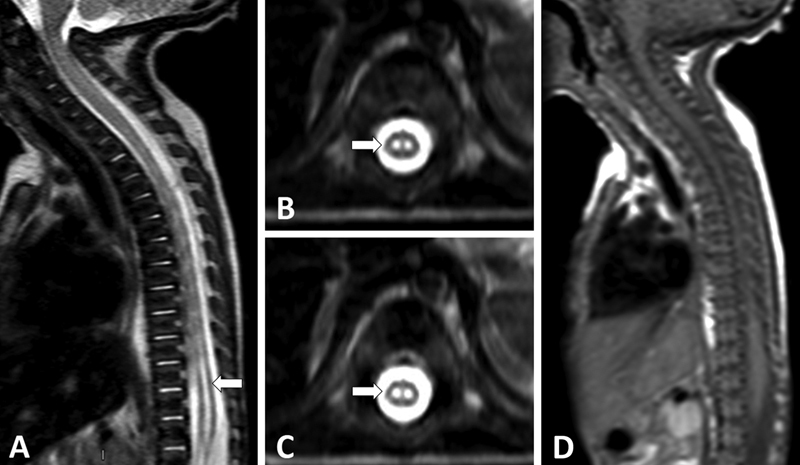
Spinal cord magnetic resonance imaging (MRI) performed at 17 days of age. Sagittal T2-weighted imaging (T2WI) shows a diffuse pencil-like hyperintense signal from D1 to the conus medullaris ((A)), and axial T2WI shows symmetric circular-ovoid foci of high signals located at the anterior horns ((B) and (C)), consistent with an “owl's eye” pattern. In its turn, sagittal T1WI ((D)) was unremarkable.

A 12-day-old male patient underwent aortoplasty for aortic arch coarctation with patent ductus arteriosus and ventricular septal defect. On the 5 ^th^ postoperative day, he presented with acute hyporeflexia, tetraparesis, and urinary retention. On spinal magnetic resonance imaging (MRI), sagittal T2-weighted image (T2WI) indicated abnormal hyperintensity extending from D1 and D2 to the conus medullaris, affecting the anterior two thirds of the spinal cord ( Figure 1A ). Additionally, axial T2WI showed the “owl's eyes” sign involving the anterior-central cord ( Figures 1B-C ) and sagital T1WI unremarkable ( Figure 1D ).

Pediatric acute spinal cord infarction is rare, and the “owl's eyes” sign on neuroimaging is highly suggestive of vascular etiology. This case is the youngest of the few ever reported in which an “owl's sign” could be observed. 1 2 3
